# Epstein-Barr virus and hepatic failure: pathogenesis, clinical manifestations, and management

**DOI:** 10.3389/fimmu.2026.1982338

**Published:** 2026-09-25

**Authors:** Wen Wang, Ying Wen

**Affiliations:** The Second Department of Infectious Diseases, The First Affiliated Hospital of China Medical University, Shenyang, Liaoning, China

**Keywords:** cytokine storm, Epstein−Barr virus, hemophagocytic lymphohistiocytosis, hepatic failure, immunopathology, immunosuppression, liver transplantation, viral load

## Abstract

Epstein-Barr virus (EBV) infection is ubiquitous, affecting over 90% of the global population, and while typically benign, it can rarely cause acute liver failure — a life-threatening complication that is frequently fatal. This review comprehensively reviews two overlapping clinical and pathogenetic patterns of EBV-associated hepatic failure — isolated severe EBV hepatitis and EBV-triggered hemophagocytic lymphohistiocytosis (HLH) — which are best understood as ends of a clinical spectrum rather than discrete entities. In isolated EBV hepatitis, liver injury is driven by CD8-positive T cells attacking EBV-infected B cells, causing bystander hepatocyte necrosis; inflammation remains confined to the liver, and supportive care is usually sufficient. By contrast, EBV-HLH involves EBV-infected monoclonal T or natural killer cells triggering a systemic cytokine storm (including interferon-gamma, tumor necrosis factor-alpha, interleukin-6, and interleukin-18) with hemophagocytosis, requiring immediate immunosuppression. In one pediatric cohort, an EBV DNA load exceeding 100,000 copies per milliliter was associated with a more than ten-fold increase in mortality, although no universally validated cutoff has been established across age groups or sample types; T-cell EBV DNA load provides additional prognostic information. Blood purification is an experimental adjunctive strategy in refractory cases, supported by very low-certainty evidence. We also discuss risk factors, diagnostic algorithms (including EBV DNA load, HLH-2004 criteria, and histopathology), therapeutic strategies (supportive care, antiviral therapy, immunomodulation, and liver transplantation), and future research priorities. Early recognition of HLH is life-saving. This review provides a practical clinical framework for differentiating these two entities and highlights knowledge gaps for future investigation.

## 1 Introduction

Epstein-Barr virus (EBV), a ubiquitous human gammaherpesvirus, is a common cause of infectious mononucleosis (IM) and is etiologically linked to various malignancies and lymphoproliferative disorders. Hepatic involvement is a frequent manifestation of acute EBV infection, typically presenting as mild, self-limited hepatitis with transient elevations in liver transaminases. In immunocompetent hosts, EBV and cytomegalovirus (CMV) are classic causes of non-hepatotropic viral hepatitis (NHVH) or acute liver failure (ALF) (1, 2). Data on NHVH remain scarce, while the epidemiology of five hepatotropic viruses — hepatitis A virus (HAV), hepatitis B virus (HBV), hepatitis C virus (HCV), hepatitis D virus (HDV), and hepatitis E virus (HEV) — is well-established (1). A systematic review estimated that viral-induced ALF has poor outcomes, and its epidemiology depends on the economic status of the studied countries (3).

Progression to ALF or acute-on-chronic liver failure (ACLF) in acute EBV infection is a rare but devastating complication (4–9). Fulminant hepatitis is observed in less than 1% of acute viral hepatitis cases, and a similar rarity applies to severe EBV-related liver failure. When it occurs, the consequences are often severe, requiring urgent liver transplantation (LT) or resulting in death (4, 8). The clinical presentation can be variable, ranging from cholestatic jaundice and marked hyperferritinemia to hemophagocytic lymphohistiocytosis (HLH) (6, 7). In patients with sickle cell disease, primary EBV infection can trigger life-threatening hepatopathy (9).

The pathogenesis of EBV-induced hepatic failure is multifactorial, involving direct viral cytopathic effects, dysregulated host immune responses, and genetic susceptibility. EBV can infect B lymphocytes and also involves T or natural killer (NK) cells in pathological contexts. Apart from direct infection of hepatocytes, the predominant mechanism in severe cases is an excessive immune response, particularly evident in overlap with HLH—a life-threatening hyperinflammatory syndrome driven by uncontrolled activation of cytotoxic T cells, NK cells, and macrophages (10, 11). In EBV-associated HLH (EBV-HLH), uncontrolled proliferation of EBV-infected T or NK cells drives massive cytokine release, causing severe liver injury. A nested case-control study found that patients with EBV-HLH had higher EBV DNA loads in T cells, which predicted HLH development and higher incidences of hepatic dysfunction (10). Genetic susceptibility, including primary immunodeficiencies such as X-linked lymphoproliferative disease (XLP), impairs the ability to control EBV, leading to fulminant IM, HLH, and fatal hepatic failure (12). Additionally, in patients with chronic liver disease (CLD), EBV DNAemia may act as a precipitant for ACLF by exacerbating liver inflammation (5).

Distinguishing isolated EBV-driven liver failure from liver failure secondary to EBV-triggered HLH is crucial, as therapeutic approaches differ significantly. Isolated EBV hepatitis, even when severe, is often managed with supportive care. However, when liver failure is a manifestation of HLH, treatment must shift to immunosuppressive and immunomodulatory therapies, including dexamethasone, etoposide and emerging agents such as emapalumab (2, 13). Novel extracorporeal therapies, such as the Seraph 100 blood filter, have been explored for pathogen removal in viral ALF, though its clinical efficacy requires further study (14). Moreover, EBV-HLH carries a high mortality if not treated aggressively, while isolated severe hepatitis may have a better outcome.

This review will provide the evidence-based mechanisms of EBV-associated hepatic failure, diagnostic hallmarks, and update treatment strategies. We synthesize current evidence from case reports, series, and emerging studies in order to optimize patient outcomes. We conducted a structured literature search of PubMed and Web of Science from inception to May 2026 using combinations of the following keywords: “Epstein-Barr virus,” “EBV,” “hepatic failure,” “acute liver failure,” “hepatitis,” “hemophagocytic lymphohistiocytosis,” “HLH,” “cytokine storm,” “immunopathology,” “viral load,” “liver transplantation,” and “immunomodulation.” Additional relevant publications were identified by hand-searching reference lists of included articles. We prioritized studies published in English, with emphasis on clinical case series, cohort studies, systematic reviews, and key molecular investigations. Given the rarity of EBV-associated hepatic failure, case reports were also included when they provided unique clinical or mechanistic insights. The final reference list was selected based on relevance to the scope of this review. This is a narrative review rather than a formal systematic review or meta-analysis; evidence is therefore synthesized qualitatively, and the strength of supporting evidence is graded throughout using a simple descriptive scheme distinguishing guideline-recommended interventions from those supported only by case series, expert opinion, or experimental data (see Tables).

## 2 Epidemiology and clinical spectrum of EBV-associated hepatic injury

### 2.1 Incidence and spectrum of liver involvement in EBV infection

Hepatic involvement is a common feature of acute EBV infection. During acute IM, up to 90% of patients exhibit some degree of liver enzyme elevation (15). This is typically characterized by a mild-to-moderate, self-limiting transaminitis, where alanine aminotransferase (ALT) and aspartate aminotransferase (AST) levels are usually less than five times the upper limit of normal (ULN) (1). It can range from anicteric hepatitis to more pronounced forms including cholestatic hepatitis especially in young adults (7). In some instances, it can lead to granulomatous hepatitis or progress to massive hepatic necrosis culminating in ALF (3). The progression to fulminant hepatic failure (FHF) or severe acute hepatitis necessitating LT is an exceedingly rare event, with an estimated incidence of less than 1% among severe EBV infections (16). This severe outcome often occurs in seemingly immunocompetent young adults or specific immunocompromised hosts, highlighting the unpredictable nature of EBV-related hepatic pathology (17). Furthermore, the epidemiological patterns of IM, which often affects younger age groups, contrast with those of classic viral hepatitis, yet both involve urban populations and can affect infants, indicating complex transmission dynamics (15). Cases progressing to ALF underscore the importance of monitoring liver function in patients with severe EBV presentations, as viral infections remain a leading cause of ALF in many regions (3). The spectrum also includes reactivation events, where EBV can cause cholestatic hepatitis even in immunocompetent individuals (18).

### 2.2 Co-existing conditions and risk factors

The clinical course and severity of EBV-related hepatic injury are significantly influenced by underlying host conditions and specific risk factors. A major predisposing factor for severe hepatic decompensation is pre-existing CLD. Acute EBV infection in individuals with chronic hepatitis B or C, or autoimmune hepatitis, can precipitate a rapid deterioration, constituting a form of ACLF (5). A higher prevalence of CMV and EBV DNAemia in patients with ACLF compared to those with stable CLD, suggests viral reactivations may act as precipitants in this vulnerable population, exacerbating liver inflammation (5). Immunosuppressed states represent another critical risk stratum. The recipients of solid organ or hematopoietic stem cell transplantation are at significant risk for severe EBV-related post-transplant lymphoproliferative disorder (PTLD), which frequently involves the liver and leads to organ failure (19). The incidence of PTLD varies by organ type and age; for example, in pediatric liver transplant recipients, the incidence is at 8.7% to 11.3%, and it has heterogeneous manifestations (20, 21). Post-transplant immunosuppression-driven uncontrolled lymphoid proliferation is the hallmark of PTLD, with the liver allograft itself being a common site of involvement in liver transplant recipients (22). Other comorbidities, such as malignancies or primary immunodeficiencies, can disrupt the delicate host-virus equilibrium, and increase uncontrolled EBV replication and subsequent severe organ damage, including hepatic failure (23). For instance, patients with inborn errors of immunity are prone to severe infections with oncoviruses like EBV, which can lead to lymphoproliferative diseases (23). Coinfection with other viruses, such as CMV, presents a higher incidence of PTLD among coinfected patients (19). In pediatric transplant patients, risk factors for PTLD include younger age at transplant, EBV seronegativity at transplantation, high post-transplant EBV viral load, and concurrent CMV disease (21, 24). Additionally, non-transplant-related immunocompromised states, such as multiple myeloma, show high susceptibility to viral infections including EBV, which can complicate their clinical course (25). Even in non-immunosuppressed hosts, co-existing conditions like sickle cell disease can act as triggers, with primary EBV infection reported to initiate acute sickle hepatopathy, leading to hepatic sequestration and life-threatening intrahepatic cholestasis (9). It is important to recognize that primary EBV infection, EBV reactivation, CAEBV, PTLD, and EBV-HLH represent distinct pathological entities with different mechanisms and management, and evidence from one setting should not be extrapolated to another without qualification.

## 3 Pathogenesis of EBV-induced hepatic failure

### 3.1 Immunological basis and target cells

The pathogenesis of EBV-associated hepatic failure is primarily immune-mediated, with liver injury resulting from a dysregulated and excessive cytotoxic immune response. EBV initially infects B lymphocytes, which triggers a massive proliferation and activation of cytotoxic CD8^+^ T cells and NK cells. These activated immune cells subsequently infiltrate the liver (26). Histologically, this process manifests as a characteristic CD8^+^ T-cell-dominated lobular hepatitis, often accompanied by sinusoidal infiltration, hepatocyte dropout, and Kupffer cell hyperplasia (27). The activated cytotoxic T cells and NK cells target EBV-infected B cells within the liver. However, the extensive hepatocyte necrosis and apoptosis observed in severe cases result from bystander effects or molecular mimicry, whereby these immune cells also attack uninfected hepatocytes and biliary epithelial cells (8). This phenomenon is exemplified in chronic active EBV (CAEBV) infection and EBV-HLH, where monoclonal or oligoclonal populations of EBV-infected T or NK cells themselves can infiltrate the liver, directly contributing to parenchymal damage and FHF (28). The specific lymphocyte subset infected by EBV differs between these diseases and correlates with clinical manifestations. In EBV-HLH, EBV infection is predominantly found in CD8^+^ T cells, whereas in CAEBV, the dominant infected populations are CD4^+^ T cells or NK cells. By contrast, in acute infectious mononucleosis, the majority of EBV-infected cells are B cells (29). These differential virus-cell interactions suggest distinct pathogenic mechanisms underlying these EBV-associated diseases and account for their different clinical presentations. The histomorphology in fatal cases typically shows centrizonal confluent hepatocytic necrosis, a portal mixed inflammatory infiltrate, and sinusoidal lymphocytosis (27).

### 3.2 Viral factors and state of EBV reactivation

The clinical severity and prognosis of EBV-associated hepatic failure are linked to viral activity. High circulating EBV DNA load, particularly reflecting lytic phase gene expression, correlates with disease severity and poor outcomes (26). Importantly, the cellular distribution of EBV DNA within lymphocyte subsets provides additional prognostic information beyond total viral load. A nested case-control study demonstrated that patients with EBV-HLH had significantly higher EBV DNA loads in T cells (median, 5.1 × 10^4^ copies/10^6^ cells) compared to those with infectious mononucleosis (median, 6.0 × 10^2^ copies/10^6^ cells). A T-cell viral load exceeding 1.5 × 10^4^ copies/10^6^ cells was associated with higher incidences of hepatic dysfunction (odds ratio 6.5), coagulation dysfunction (odds ratio 15.7), and other organ dysfunctions, and increased the risk of developing EBV-HLH (hazard ratio 4.7) (10). It is important to note that EBV pathogenicity depends not only on viral load but also on its complex latency state. Different latency programs (e.g., Latency I, II, III) are defined by distinct viral gene expression profiles (e.g., Latent Membrane Proteins, EBV Nuclear Antigens), which profoundly influence host immune recognition and response, thereby shaping the disease pathology (30). Monitoring this viral load via quantitative polymerase chain reaction (PCR) becomes the cornerstone for diagnosis and monitoring of liver failure, as traditional serological profiles can be atypical or delayed. For instance, antibodies against viral capsid antigen (VCA-IgM), early antigen (EA-D), and Epstein-Barr nuclear antigen (EBNA-1 IgG) may not follow classic patterns in severe cases, limiting their immediate diagnostic utility (31). The direct cytopathic effect of EBV on hepatocytes is generally considered less significant than immune-mediated damage, but may play a more prominent role in immunocompromised individuals or in biliary epithelial cells (32). In CAEBV, persistent high-level viremia and the clonal expansion of EBV-infected T or NK cells (rather than B cells) are key pathogenic features of severe hepatitis and HLH (33). Genotyping or genomic analysis of EBV in such cases can reveal sequence variations in genes related to specific latency programs or cell tropism (e.g., genes involved in packaging, latency, or structural proteins like BPLF1 and LMP1), providing molecular insights into viral strain diversity and potential pathogenic variants (34). Furthermore, latent EBV reactivation, triggered by other infections or immunosuppression, can precipitate severe hepatic injury, as seen in post-transplant settings or other immunodeficiencies (35).

A major limitation of current evidence is the lack of standardization of EBV DNA quantification. Reported thresholds for “high” viral load vary widely across studies, and direct comparison is further hampered by differences in sample type — whole blood, plasma, and peripheral blood mononuclear cells (PBMCs) yield different quantitative results — as well as by inter-assay variability and the absence of an international calibrator (10, 30, 31). Consequently, no universally validated EBV DNA cutoff for predicting hepatic failure or mortality has been established, and any threshold cited in this review (for example, 10^4^ or 10^5^ copies/mL) should be interpreted within the specific study context. Whenever possible, serial measurements on a consistent sample type and matrix are preferable to single values, and cellular EBV DNA load (for example, in T cells) may provide additional disease-specific prognostic information beyond total viral load (10).

Regarding EBV strain diversity, the virus comprises two major types (type 1 and type 2) that differ in the EBNA-2/EBNA-3 gene region, together with numerous naturally occurring variants across genes such as LMP1, BZLF1, and BPLF1 (36). Emerging data suggest that specific EBV variants and gene polymorphisms may influence viral latency, cell tropism, oncogenic potential, and possibly the severity of EBV-associated disease, including hepatic involvement (34, 36, 37). However, the clinical significance of most sequence variations remains incompletely defined, and genotyping is not currently part of routine management. Of potential clinical relevance, EBV type 2 preferentially infects T lymphocytes, whereas type 1 transforms B cells more efficiently (38, 39); type 2 infection has also been associated with higher viral loads than type 1 in some cohorts (40, 41). Given that EBV-HLH is driven by EBV infection of T or NK cells and that high viral load correlates with severe hepatic disease, whether type 2 strains confer a higher risk of HLH-type liver failure warrants dedicated investigation. Larger, standardized studies correlating EBV genotype with clinical phenotype in hepatic failure are needed before strain typing can inform prognosis or therapy.

### 3.3 Genetic predisposition and syndromic associations

Individual susceptibility to severe EBV hepatitis and hepatic failure is influenced by genetic factors that govern immune response and lymphocyte cytotoxicity. Monogenic disorders affecting genes controlling lymphocyte cytotoxicity, such as *PRF1*, *UNC13D*, and *STX11* associated with familial HLH, predispose individuals to develop HLH following EBV infection, with hepatic failure as a severe manifestation (42). Primary immunodeficiencies such as XLP types 1 and 2 confer a high risk of fatal EBV infection with massive hepatic involvement, often presenting as fulminant IM (FIM) and rapid progression to liver failure (12, 43). Beyond these severe syndromic associations, polygenic variations in immune response genes also modulate this risk. Specific human leukocyte antigen (HLA) alleles are associated with both susceptibility to severe EBV-related diseases and the nature of the immune response. For example, certain HLA haplotypes are linked to primary sclerosing cholangitis (PSC) and are also restricted for EBV epitopes, suggesting EBV-specific T cell responses in disease pathogenesis (44). Similarly, HLA associations influence antibody responses to EBV antigens like EBNA-1, which are linked to disease risk in multiple sclerosis, highlighting the complex interplay between genetics and antiviral immunity (45). Other genetic defects, such as mutations in *NFAT5* leading to haploinsufficiency, or mutations in *MAGT1* cause X-linked immunodeficiency with magnesium defect, EBV infection, and neoplasia (XMEN) disease, as well as fatal EBV infections (42, 46).

### 3.4 Inflammatory mediators and the role of HLH

The transition from severe EBV hepatitis to FHF is driven by a dysregulated release of pro-inflammatory cytokines, often termed a “cytokine storm.” Key mediators include interferon-gamma (IFN-γ), tumor necrosis factor-alpha (TNF-α), interleukin-6 (IL-6), and interleukin-18 (IL-18), which promote hepatocyte apoptosis, coagulopathy, and multi-organ dysfunction (47, 48). This hyperinflammatory state underlies the pathogenesis of HLH, a severe end of EBV-related disease. The pathophysiological continuum between severe EBV hepatitis and HLH is critical; some patients with EBV-hepatic failure meet only part of the HLH diagnostic criteria, representing an “incomplete” or “forme fruste” HLH (49, 50). In “isolated” severe EBV hepatic failure, liver damage may primarily result from an intense cytotoxic T-cell response localized within the liver. In contrast, HLH-associated hepatic failure arises from systemic immune dysregulation characterized by impaired cytotoxic function of NK and T cells, uncontrolled immune activation, and widespread hemophagocytosis involving the liver, bone marrow, spleen, and other organs (27, 51). This systemic cytokine storm can be targeted therapeutically, for example with the anti-IFN-γ monoclonal antibody emapalumab in treating refractory EBV-HLH (47). The detection of hemophagocytic figures in the liver, spleen, and bone marrow is a hallmark of this process in fatal cases (27). Therefore, recognizing HLH and its inflammatory mediators dictates a need for immunosuppressive or immunomodulatory therapies beyond mere antiviral or supportive care for EBV-induced liver failure. The key differences in pathogenesis between isolated severe EBV hepatitis and EBV-HLH-driven hepatic failure are summarized in **Figure 1**.

**Figure 1:**
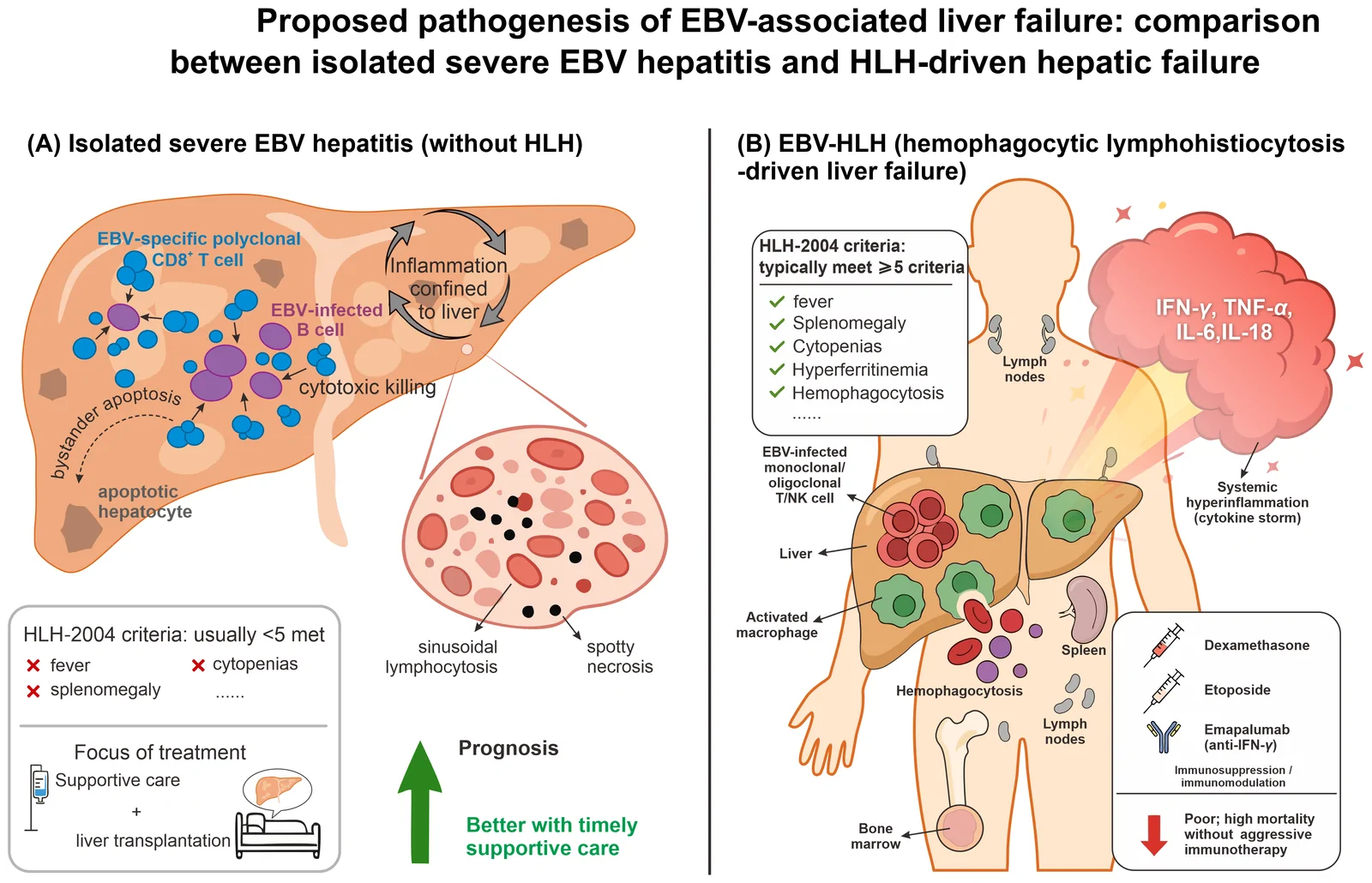
EBV-associated liver failure: isolated severe hepatitis vs EBV-HLH-driven hepatic failure. (conceptual model; the two patterns represent ends of a clinical spectrum with potential overlap, rather than mutually exclusive categories). **(A)** Isolated severe EBV hepatitis (without HLH). The pathogenesis is centered on the liver. EBV-infected B cells (purple) are targeted by EBV-specific polyclonal CD8^+^ T cells (blue), leading to bystander apoptosis of adjacent hepatocytes (brown). Histological features include sinusoidal lymphocytosis and spotty necrosis, typically without significant hemophagocytosis. Inflammation is predominantly confined to the liver, although mild systemic features may occur. Patients typically meet fewer than five HLH-2004 criteria, and management focuses on supportive care with consideration of liver transplantation. Prognosis is relatively favorable with timely supportive therapy. **(B)** EBV-HLH-driven hepatic failure. The disease involves systemic hyperinflammation. EBV-infected monoclonal/oligoclonal T or NK cells (red) and activated macrophages (green) infiltrate multiple organs (liver, spleen, bone marrow, lymph nodes). Hemophagocytosis (engulfment of blood cells by macrophages) is present. A cytokine storm (IFN-γ, TNF-α, IL-6, IL-18) spreads systemically, driven by impaired NK/CTL cytotoxicity. Patients typically meet five or more HLH-2004 criteria. Treatment requires immunosuppression/immunomodulation (dexamethasone, etoposide, emapalumab). Prognosis is poor without aggressive immunotherapy.

## 4 Clinical manifestations and diagnostic approach

### 4.1 Hepatic and systemic clinical features

The hepatic presentation of EBV-associated ALF closely mirrors that of ALF from other etiologies, characterized by a rapid onset of jaundice, coagulopathy with an elevated international normalized ratio (INR), hepatic encephalopathy, and ascites (2). A preceding prodrome of fever, pharyngitis, and lymphadenopathy, typical of IM, may be present, serving as an initial clue to the underlying viral cause (52). However, the clinical picture is often dominated by a systemic inflammatory process, which includes persistent fever, splenomegaly, and cytopenias, particularly thrombocytopenia (53). This constellation of systemic symptoms is highly indicative of concomitant HLH (54). Distinguishing EBV-induced hepatic failure from HAV, HBV or HEV-induced hepatic failure is crucial. A key hematological differentiator is the marked atypical lymphocytosis on peripheral blood smear, a hallmark of EBV infection (6). Furthermore, diagnosis relies on specific serological and virological markers, such as positive EBV immunoglobulin M (IgM) antibodies or a high EBV DNA load in the blood, especially in severe cases (53). Some patients present with the classic IM triad, whereas others, particularly immunocompetent older adults, may exhibit an atypical presentation with severe cholestatic hepatitis and marked hyperbilirubinemia, even in the absence of typical IM symptoms like pharyngitis or lymphadenopathy (55). This underscores considering EBV in the differential diagnosis of acute cholestatic hepatitis of unknown origin (56). The hepatic injury itself can range from mild, self-limited transaminitis to FHF with massive necrosis (57). In severe cases, the liver failure can progress rapidly to multi-organ failure and death, especially with delayed diagnosis and an underlying immunodeficiency (43). DILI (e.g., from acetaminophen or antithyroid drugs) or co-infections (e.g., HAV, CMV, or SARS-CoV-2), can compound the hepatic injury and create diagnostic confusion, presenting a significant clinical challenge (11, 58, 59). For clarity, we use the following definitions throughout: acute liver injury (ALI) refers to acute hepatocellular damage (elevated aminotransferases) without coagulopathy or encephalopathy; ALF requires jaundice, coagulopathy (INR ≥1.5), and hepatic encephalopathy of any degree, developing within 26 weeks in a patient without pre-existing cirrhosis; FHF denotes the most rapid and severe end of this spectrum; and ACLF refers to acute decompensation superimposed on CLD (2, 3).

### 4.2 Diagnostic criteria and key investigations

In patients presenting with ALF, EBV status should be considered especially when accompanied by systemic symptoms suggestive of IM or HLH. The cornerstone of diagnosis is demonstration of active EBV infection, typically indicated by a high EBV DNA load (often >10^4^ copies/mL) in peripheral blood in severe disease (8, 53). The diagnostic approach must be systematic. First, it requires the confirmation of ALF based on standard clinical criteria: acute onset of liver injury, jaundice, coagulopathy (INR ≥1.5), and hepatic encephalopathy occurring within 26 weeks in a patient without pre-existing cirrhosis (2). Key laboratory investigations beyond liver function tests should include a complete blood count to identify atypical lymphocytosis and cytopenias (a hallmark of HLH), and measurement of ferritin and soluble interleukin-2 receptor (sCD25) levels, both of which are markedly elevated in HLH (54, 60). Although cytokine profiling is increasingly recognized as a valuable tool for the diagnosis and management of HLH, robust evidence supporting its specific diagnostic correlations remains limited (61). Screening for HLH should use HLH-2004 criteria, which include fever, splenomegaly, cytopenias, hypertriglyceridemia and/or hypofibrinogenemia, hemophagocytosis in bone marrow/spleen/lymph nodes, low or absent NK cell activity, and elevated ferritin and sCD25 (54). A critical step in the diagnostic workup is to exclude other common causes of ALF. This involves comprehensive serological testing for hepatotropic viruses (HAV, HBV, HCV, HEV), autoimmune markers (anti-nuclear antibody, anti-smooth muscle antibody, etc.), and a thorough review for potential DILI (3, 62). When clinically feasible and safe given the coagulopathy, a liver biopsy can provide invaluable diagnostic information. Characteristic histopathological findings can support the diagnosis and help rule out other conditions like autoimmune hepatitis or lymphoma that may mimic EBV hepatitis (63). The diagnostic process is further complicated in patients with underlying CLD, where EBV reactivation can precipitate ACLF (5). In such scenarios, EBV DNAemia may represent a previously unrecognized precipitant. Based on the available evidence and our clinical experience, we propose a practical diagnostic algorithm for patients with suspected EBV-associated hepatic failure: (i) all patients with ALF of unclear etiology should undergo EBV DNA PCR testing; (ii) in those with detectable EBV DNA, a complete blood count, ferritin, triglycerides, fibrinogen, and sCD25 should be measured to screen for HLH; (iii) if ≥4 HLH-2004 criteria are met (rather than waiting for all 5), early consultation with hematology and empirical immunomodulatory therapy may be considered. This lower threshold is based primarily on expert opinion and has not been prospectively validated, and it should be applied with caution, particularly because ALF itself can produce cytopenia, hyperferritinemia, and hypofibrinogenemia that overlap with HLH features, and because tools such as the HScore are increasingly used in adults with suspected HLH. A practical summary of diagnostic criteria and key investigations is provided in **Table 1**.

**Table 1:** Diagnostic key points for EBV-associated liver failure.

Diagnostic element	Key points
Clinical features (2, 54)	Acute liver failure (ALF): jaundice, INR ≥1.5, encephalopathy within 26 weeks without prior cirrhosis; may have fever, pharyngitis, lymphadenopathy. Persistent high fever, splenomegaly, cytopenias → suspect HLH.
Virologic confirmation (8, 53, 79)	Quantitative EBV DNA PCR on peripheral blood is a central diagnostic tool, but should be interpreted with the sample type and assay in mind; no universally validated cutoff exists. High viral load (often >10
HLH screening (54, 60)	Meet ≥5 of HLH‑2004 criteria: fever, splenomegaly, cytopenias (≥2 lineages), hypertriglyceridemia/hypofibrinogenemia, hemophagocytosis, low/absent NK cell activity, hyperferritinemia, elevated sCD25. In adults, the HScore may complement HLH-2004 criteria; note that ALF itself can produce cytopenia, hyperferritinemia, and hypofibrinogenemia that overlap with HLH.
Exclusion of other causes (3, 62)	Hepatotropic viruses (HAV, HBV, HCV, HEV), autoimmune hepatitis, drug‑induced liver injury, other viruses (CMV, HSV, adenovirus).
Liver biopsy (if feasible) (8, 27, 63)	CD8^+^ T‑cell‑predominant lobular hepatitis, sinusoidal lymphocytosis, hemophagocytosis; EBER *in situ* hybridization positive in infiltrating lymphocytes (especially T cells).
Genetic evaluation (12, 42, 54)	Consider X‑linked lymphoproliferative disease (XLP), familial HLH genes (PRF1, UNC13D, STX11), especially in children or positive family history.

EBV, Epstein-Barr virus; ALF, acute liver failure; INR, international normalized ratio; HLH, hemophagocytic lymphohistiocytosis; PCR, polymerase chain reaction; VCA, viral capsid antigen; IgM, immunoglobulin M; EA, early antigen; EBNA, Epstein-Barr nuclear antigen; NK, natural killer; sCD25, soluble CD25 (soluble interleukin-2 receptor); CMV, cytomegalovirus; HSV, herpes simplex virus; EBER, Epstein-Barr virus-encoded small RNA; XLP, X-linked lymphoproliferative disease.

### 4.3 Histopathological correlates and literature synthesis

Histopathological examination of the liver, when obtainable, can reflect the underlying immunopathological process. The hallmark is a dense portal and lobular inflammatory infiltrate predominantly composed of CD8^+^ T lymphocytes (8). These virus-specific cytotoxic T cells are implicated in both controlling the infection and mediating hepatocyte injury. The parenchymal injury manifests as spotty (apoptotic) to confluent hepatocyte necrosis, the extent of which correlates with clinical severity, ranging from mild hepatitis to massive hepatic necrosis in fulminant cases (57). A distinctive feature is sinusoidal lymphocytosis, where lymphocytes are seen within the hepatic sinusoids, a pattern that can sometimes be confused with intravascular lymphoma (64). Kupffer cell hypertrophy and hyperplasia are commonly observed, and in cases of HLH, active hemophagocytosis—where Kupffer cells engulf erythrocytes, leukocytes, or platelets—may be evident within the sinusoids (60). Notably, cholestasis is often minimal relative to the degree of inflammation and hepatocellular injury, helping to distinguish it from primary biliary disorders (2). In severe or atypical cases, *in situ* hybridization for EBV-encoded small RNA (EBER) can be performed on liver tissue. Positivity within infiltrating lymphocytes, particularly within monoclonal CD8^+^ T cells, indicates direct viral involvement and a more liver-restricted hyperinflammatory state that may recur even after LT, necessitating systemic chemotherapy and hematopoietic cell transplantation for cure (8). The characteristic histopathological findings of EBV-associated liver failure are summarized in **Table 2**.

**Table 2:** Pathological features of EBV-associated liver failure.

Pathological feature	Description
Inflammatory infiltrate (8, 63)	Portal and lobular infiltrate dominated by CD8^+^ T lymphocytes; plasma cells less prominent than in autoimmune hepatitis.
Hepatocellular necrosis (27, 57)	Spotty (apoptotic) to confluent necrosis; in fulminant cases, massive or submassive necrosis.
Sinusoidal lymphocytosis (64)	Lymphocytes within hepatic sinusoids; should not be mistaken for intravascular lymphoma.
Kupffer cell changes (27, 60)	Hypertrophy and hyperplasia; active hemophagocytosis (engulfment of erythrocytes, leukocytes, platelets) – hallmark of HLH involvement.
Cholestasis (2)	Usually mild and disproportionate to the degree of hepatocellular injury; helps differentiate from primary biliary disorders.
EBER *in situ* hybridization (8)	Positive in infiltrating lymphocytes; monoclonal CD8^+^ T‑cell positivity indicates aggressive, liver‑restricted hyperinflammatory state (e.g., chronic active EBV).
Cholestatic variant (7, 55)	Rare but can present with severe cholestasis and hyperbilirubinemia even without typical IM symptoms; no specific diagnostic histology except bile plugs.

EBV, Epstein-Barr virus; CD8, cluster of differentiation 8; EBER, Epstein-Barr virus-encoded small RNA; IM, infectious mononucleosis.

This histopathological profile helps differentiate EBV hepatitis from other conditions, such as autoimmune hepatitis (which typically shows a plasma cell-rich interface hepatitis) or DILI (63). Synthesizing literature from key case series and studies underscores the grave prognosis of EBV infection when complicated by HLH and liver failure (54). Patient demographics often include both immunocompetent and immunocompromised hosts, with children and young adults being frequently affected, though severe cases in older adults are also documented (55). Reported EBV DNA levels in severe disease are typically very high. Treatment regimens are multifaceted, often involving a combination of supportive care for ALF, antiviral agents (though their efficacy in established ALF is debated), and aggressive immunosuppressive/immunomodulatory therapy targeting HLH, such as corticosteroids, etoposide, and intravenous immunoglobulin (IVIG) based on the HLH-94/2004 protocols (60, 65). The outcomes are particularly dismal in patients with underlying immunodeficiencies, such as XLP (12, 43). Furthermore, co-infections with other viruses (e.g., CMV, adenovirus) or concurrent DILI can exacerbate liver damage and confound both diagnosis and management (58, 66). A summary of published cases of EBV-HLH presenting with hepatic failure is shown in **Table 3**.

**Table 3:** Literature summary of EBV-associated HLH with hepatic failure.

First author, year	Patient age/underlying condition	EBV evidence	Liver failure presentation	Main treatment	Outcome
Hattori A, 2021 (60)	Adult, immunocompetent	EBV DNA positive	Acute liver injury (elevated ALT)	Etoposide + dexamethasone	Recovery
Nakajima K, 2022 (8)	Child	High EBV DNA, EBER^+^ CD8^+^ T cells in liver	ALF requiring liver transplant	LT → chemotherapy → HSCT	Cure
Mitra S, 2024 (27)	Adult, immunocompetent (autopsy)	EBV serology & PCR	ALF with hemophagocytosis in liver	Supportive care	Death
Wang W, 2024 (47)	Case series (EBV‑HLH)	EBV DNA positive	Hepatic dysfunction	Emapalumab (anti‑IFN‑γ)	Improvement
Iizuka H, 2021 (51)	Adult with EBV^+^ DLBCL	EBV-positive diffuse large B‑cell lymphoma	Liver failure, hemophagocytosis	Chemotherapy	Death (autopsy)
Sun FY, 2023 (65)	Adult	Elevated EBV DNA	Acute liver injury + HLH	Ganciclovir + dexamethasone + etoposide	Recovery
Dosanjh HS, 2025 (7)	Young adult	Acute EBV hepatitis	Severe cholestatic hepatitis, secondary HLH	Supportive care	Recovery
Yan H, 2025 (79)	Children (cohort)	EBV DNA >1×10^5^ copies/mL	Liver injury + HLH	Not detailed (cohort study)	Higher mortality (OR = 10.53)
Chen C, 2026 (13)	Post‑liver transplant	EBV reactivation	Severe liver dysfunction in HLH	Emapalumab	Improvement

EBV, Epstein-Barr virus; HLH, hemophagocytic lymphohistiocytosis; DNA, deoxyribonucleic acid; ALT, alanine aminotransferase; ALF, acute liver failure; LT, liver transplantation; HSCT, hematopoietic stem cell transplantation; DLBCL, diffuse large B-cell lymphoma; OR, odds ratio. Inclusion criteria for **Table 3**: cases were included if they described EBV-associated HLH with clinically significant liver involvement. We use “acute liver injury” to denote elevated aminotransferases without documented coagulopathy or encephalopathy, “severe acute hepatitis” for pronounced hepatocellular injury that did not fulfill formal ALF criteria, and “acute liver failure (ALF)” only when coagulopathy (INR ≥1.5) with encephalopathy was documented. Cases with only mild or transient transaminitis were excluded.

The clinical profile of EBV-associated hepatic failure differs importantly between children and adults. Primary EBV infection predominates in children and adolescents, in whom severe EBV hepatitis and EBV-HLH frequently occur against a background of previously unrecognized genetic predisposition, such as familial HLH genes (PRF1, UNC13D, STX11) or XLP disease (12, 42). Pediatric EBV-HLH is typically triggered by primary infection and is strongly associated with clonal proliferation of EBV-infected T or NK cells, whereas adult cases more often arise from EBV reactivation, underlying hematological malignancy (including EBV-positive lymphoma), or iatrogenic immunosuppression (51, 54). Older immunocompetent adults may present atypically with severe cholestatic hepatitis and marked hyperbilirubinemia in the absence of classic infectious mononucleosis features, which can delay diagnosis (55). These differences have direct implications: genetic evaluation and early consideration of primary HLH are priorities in pediatric cases, whereas adults require a focused search for occult malignancy and review of immunosuppressive medications.

Diagnosis is substantially more difficult in patients with pre-existing liver disease or immunosuppression. In patients with cirrhosis or CLD, EBV DNAemia may represent a precipitant of ACLF rather than the primary driver of injury, and baseline coagulopathy, cytopenia, and hyperferritinemia can obscure superimposed HLH (5). In solid-organ or hematopoietic stem-cell transplant recipients, EBV infection spans a continuum from asymptomatic DNAemia to PTLD and EBV-HLH, and these distinct entities must be carefully separated because their management differs; serial viral-load monitoring and EBER *in situ* hybridization on involved tissue are central to this distinction (19, 22, 67). Profoundly immunosuppressed patients may mount atypical or delayed antibody responses, so serology alone is insufficient, and quantitative EBV DNA together with infected-cell subset analysis should be prioritized (31, 32).

## 5 Therapeutic management strategies

### 5.1 Foundational supportive care and liver transplantation

The management of EBV-associated hepatic failure adheres to the general principles of ALF protocols, which are centered on intensive care support and the management of life-threatening complications (68). This includes meticulous correction of coagulopathy, hepatic encephalopathy and cerebral edema, and secondary infections (68).

In patients with HLH-associated hepatic failure who fail to respond promptly to immunomodulatory therapy, blood purification modalities may serve as an experimental adjunctive strategy, supported by very low-certainty evidence. Direct evidence specifically for blood purification in EBV-HLH with liver failure is limited, but accumulating data from HLH of other etiologies suggest a potential benefit. Therapeutic plasma exchange (TPE) has been reported to improve clinical and laboratory parameters in pediatric HLH refractory to HLH-2004 regimens (69) and to reverse severe HLH induced by immune checkpoint inhibitors (70); however, TPE itself has occasionally been associated with reactive hemophagocytosis (71). A combination of plasma exchange with continuous renal replacement therapy (PE+CRRT) was well tolerated in eight children with severe EBV-associated HLH, leading to normalization of most cytokines, reduction of EBV DNA and ferritin, and sustained complete remission in seven cases (72). A recent retrospective study of 102 pediatric HLH patients demonstrated that early addition of blood purification to HLH-94/04 protocols significantly improved survival, particularly in those with multi-organ dysfunction or poor early response (73). Continuous hemofiltration (CHF) has also been used to stabilize metabolic status in children with HLH-induced multi-organ failure (74), and hemoperfusion with a polymyxin B column resulted in rapid recovery in a case of lymphoma-associated HLH unresponsive to chemotherapy (75). Beyond HLH, blood purification has been explored for direct pathogen removal in EBV-associated ALF: an exploratory study using the Seraph 100 blood filter together with therapeutic plasma exchange demonstrated significant clearance of EBV DNA (14). Taken together, while most evidence comes from case reports or small series and is not specific for EBV-HLH with hepatic failure, these observations indicate that blood purification can reduce circulating cytokines and, in some cases, viral load, thereby potentially ameliorating hyperinflammation and liver injury. This approach may serve as a bridge to definitive therapies such as immunosuppressive regimens or LT in critically ill patients.

Given the high mortality, early referral to a specialized liver transplant center is paramount (68). The decision of LT requires a careful assessment of the potential for spontaneous recovery versus the risk of irreversible hepatic necrosis (8). In this context, a high EBV DNA load in the serum often serves as a negative prognostic marker, indicating a lower likelihood of recovery without intervention and underscoring the need for expedited transplant evaluation (8). Post-transplant management requires sustained vigilance to prevent and treat EBV reactivation and its severe consequence including PTLD in the new graft (76). EBV infection is a major cause of PTLD, which can lead to graft failure and increased mortality (76). Monitoring EBV viral load post-transplant indicates that a high viral load, especially in EBV-seronegative recipients, is a significant risk factor for developing PTLD (67).

### 5.2 Targeting the virus: antiviral therapies

The role of specific antiviral therapies, such as ganciclovir or valganciclovir, in the treatment of EBV-associated hepatic failure remains controversial and is not well-supported by robust clinical evidence (1). This is primarily because the severe liver injury in many cases, particularly those progressing to ALF, is driven by an intense immune-mediated response—such as HLH —rather than direct viral cytopathic effect (8). Consequently, antiviral agents are generally considered adjunctive rather than curative. Their use may be more rationally considered in specific subgroups, such as profoundly immunosuppressed patients or those with high viral loads, where controlling viral replication might theoretically mitigate immune activation (1). The evidence for nucleoside analogues, which inhibit viral DNA polymerase, is largely limited to anecdotal reports (1). Newer antiviral agents like maribavir, which targets the UL97 kinase, are under investigation for resistant EBV infections, but data on their efficacy in the setting of life-threatening liver failure are currently lacking (1). A critical principle in management is that antiviral therapy should not delay the initiation of immunomodulatory treatment if features of HLH or severe hyperinflammation are present, as addressing the dysregulated immune response is often life-saving (60).

### 5.3 Immunomodulation: core of therapy for immune-mediated injury

For patients with EBV-associated hepatic failure who exhibit features of HLH or severe systemic hyperinflammation, immunomodulatory therapy forms the cornerstone of life-saving intervention (60). The first-line approach typically follows established HLH protocols, such as the HLH-94/2004 regimen, which combines dexamethasone with etoposide to suppress the hyperactive immune system and eliminate activated lymphocytes (60). This strategy is supported by case reports where such treatment led to the resolution of life-threatening liver injury secondary to EBV-HLH (60). IVIG may provide benefit by neutralizing viral particles and modulating the broader immune response, though its use is often integrated into a multi-agent strategy (77). Cyclosporine A, which inhibits T-cell activation, is another option within the HLH therapeutic framework (77). For cases refractory to first-line HLH therapy, emerging salvage options include targeted anti-cytokine therapies, such as the interleukin-1 receptor antagonist anakinra, the IL-6 receptor antagonist tocilizumab, and the interferon-γ monoclonal antibody emapalumab, which aim to quell the specific cytokine storms driving tissue damage (77). However, their use in EBV-triggered liver failure is extremely limited, and their application should be considered experimental within this specific context (77). Furthermore, caution is warranted as immunomodulation itself, particularly with agents like tocilizumab, has been associated with exacerbations of underlying viral hepatitis (78). An overview of antiviral and immunomodulatory drugs used in EBV-associated liver failure, including their mechanisms and evidence levels, is presented in **Table 4**.

**Table 4:** Summary of antiviral and immunomodulatory drugs for EBV-associated liver failure.

Drug class	Drug name	Mechanism	Main indication in EBV liver failure	Evidence level
Antiviral (1, 2)	Ganciclovir/Valganciclovir	Inhibits EBV DNA polymerase	Immunocompromised patients or very high viral load; not routinely recommended because liver injury is mainly immune‑mediated.	Expert opinion, case reports
Antiviral (1)	Maribavir	Inhibits UL97 kinase (approved for refractory/resistant CMV, not for EBV)	No approved indication for EBV; very limited experimental data; not recommended for EBV-associated liver failure.	Not recommended
Immunosuppression (HLH regimen) (54, 60)	Dexamethasone	Broad anti‑inflammatory, suppresses cytokines	First‑line for HLH‑associated liver failure (HLH‑94/2004 protocol).	Guideline‑recommended
Immunosuppression (HLH regimen) (54, 60, 65)	Etoposide	Eliminates activated T cells and macrophages	Core drug for EBV‑HLH to control cytokine storm.	Guideline‑recommended
Calcineurin inhibitor (77)	Cyclosporine A	Inhibits T‑cell activation	Alternative or adjunct in HLH protocols.	Optional
Biologic (anti‑cytokine) (13, 47)	Emapalumab (anti‑IFN‑γ mAb)	Neutralizes interferon‑gamma	Refractory EBV‑HLH with severe liver injury; shown promise in post‑transplant setting.	Case reports/small series
Biologic (anti‑IL‑1) (77)	Anakinra (IL‑1 receptor antagonist)	Blocks IL‑1 signaling	Salvage therapy for HLH/MAS; minimal data specifically for EBV‑liver failure.	Experimental
Biologic (anti‑IL‑6) (77, 78)	Tocilizumab (IL‑6 receptor antagonist)	Blocks IL‑6 signaling	Same as above; caution – may exacerbate viral hepatitis.	Experimental, cautious
Passive immunity (77)	Intravenous immunoglobulin (IVIG)	Immunomodulation, viral neutralization	Adjunctive therapy, often combined with other agents.	Expert opinion
Anti‑CD20 (54)	Rituximab	Depletes EBV‑infected B cells	Mainly for EBV‑associated PTLD; occasionally used in HLH to reduce B‑cell drive.	Selected cases

EBV, Epstein**-**Barr virus; HLH, hemophagocytic lymphohistiocytosis; MAS, macrophage activation syndrome; DNA, deoxyribonucleic acid; IFN**-**γ, interferon**-**gamma; IL, interleukin; TNF**-**α, tumor necrosis factor**-**alpha; mAb, monoclonal antibody; PTLD, post**-**transplant lymphoproliferative disorder; NK, natural killer; CTL, cytotoxic T lymphocyte; IVIG, intravenous immunoglobulin; CD20, cluster of differentiation 20.

Regarding emerging therapies, data on newer agents in EBV-associated hepatic failure remain limited and largely derive from case reports and small series. Emapalumab, an anti-interferon-γ monoclonal antibody approved for primary HLH, has been reported to improve refractory EBV-HLH and severe post-transplant liver dysfunction in selected cases (13, 47); however, no randomized trial has evaluated its efficacy or safety specifically in EBV-HLH with hepatic failure, and its use is constrained by high cost and restricted availability. Anakinra and tocilizumab have shown benefit in some HLH/macrophage activation syndrome settings, but evidence in EBV-related disease is minimal, and tocilizumab carries a caution for potential exacerbation of viral hepatitis (77, 78). Ruxolitinib and other JAK inhibitors are of growing interest given their cytokine-suppressive activity, but are experimental in this context. Detailed outcome data — including mortality, time to response, and toxicity — are lacking, underscoring the need for prospective, multicenter evaluation before these agents can be recommended outside clinical trials or compassionate use.

In our clinical practice, the decision to initiate HLH-directed therapy is often more time-critical than the decision to confirm the diagnosis definitively. For patients with suspected EBV-HLH and progressive liver dysfunction, starting dexamethasone (10 mg/m^2^/day) may be considered while awaiting confirmatory test results, as this approach carries relatively low risk and may be life-saving. For those who do not respond within 48–72 hours, the early addition of etoposide may be considered after multidisciplinary assessment, guided by the EBV-infected cell type determined through lymphocyte subset sorting (e.g., T/NK-cell versus B-cell tropism). We emphasize that these suggestions reflect our institutional practice and expert opinion rather than evidence-based recommendations; treatment strategies for adult EBV-HLH vary substantially among institutions, and etoposide carries important toxicity risks in patients with severe hepatic dysfunction. Although emapalumab shows promise in refractory cases, its high cost and limited availability currently restrict its use to specialized centers in our region.

## 6 Prognosis and outcomes

### 6.1 Prognostic determinants

The prognosis of EBV-associated hepatic failure is determined by a combination of virological, clinical, and genetic factors. The most important laboratory prognostic factor is EBV DNA load in peripheral blood. A large-scale study investigating active EBV infection in children found a positive correlation between EBV DNA levels and mortality rate, as well as the incidence of liver injury and other severe complications such as acute kidney injury and coagulation abnormalities (79). The risk of mortality significantly increased when the EBV DNA load exceeded 1 × 10^5^ copies/mL, with an adjusted odds ratio of 10.53; however, this threshold was derived from a pediatric cohort and should not be generalized as a universally applicable cutoff for adult EBV-HLH or EBV-associated hepatic failure (79). In adult secondary HLH (sHLH), patients with higher pre-treatment plasma EBV-DNA loads (>8.51×10^4^ copies/mL) had worse overall survival compared to those with lower loads or negative results (80). Persistently high or rising viral titers are associated with mortality and the need for LT (52). When associated with EBV, the development of full-blown HLH confers a worse prognosis compared to isolated severe hepatitis (11). Cases of EBV-HLH present with rapid impairment of liver function and coagulation, even multiple organ failure (51, 54). The prognosis is dismal when HLH is triggered by underlying malignancies, such as EBV-positive diffuse large B-cell lymphoma (51). Genetic screening for HLH-associated mutations can identify patients at risk for severe disease and inform family counseling. Primary HLH (pHLH) can be driven by genetic defects in genes such as those involved in familial HLH (e.g., PRF1, UNC13D), XLP disease (e.g., SH2D1A, XIAP), and other EBV susceptibility diseases (54). Identifying these mutations is crucial, as primary immunodeficiencies like XLP disease are unable to control EBV infection and are prone to develop fulminant hepatitis and lymphoma (12).

### 6.2 Long-term outcomes and survivorship

Long-term outcomes of EBV-associated hepatic failure vary significantly based on disease severity and therapeutic intervention. Survivors who do not require transplantation may recover with normal or near-normal liver function, though comprehensive long-term data are sparse. EBV-associated self-limited acute hepatitis often resolves with complete hepatic recovery (52). In a study of acute viral hepatitis, ALF was rare (1.3%), and no mortality or LT was reported (81). However, some patients may experience cholestatic hepatitis or progress to chronic hepatitis (52). Patients who undergo LT face immunosuppression management and EBV reactivation. Infection with non-hepatotropic viruses like EBV remains an important cause of graft failure and rejection after LT (76). Prophylactic strategies, such as the use of anti-CMV hyperimmunoglobulin (CMVIg), can reduce the incidence of severe immunological and septic events, including EBV infections (82). PTLD, as illustrated by a case of late-onset HLH in a lung transplant patient, proved fatal despite treatment (83). For pediatric patients with CAEBV, allogeneic hematopoietic stem cell transplantation (HSCT) is the only curative measure, but it carries risks of post-transplant complications (84). Neurological and quality-of-life outcomes in survivors represent an important area for future study. Neurological involvement, such as encephalopathy, is a recognized poor prognostic sign in ALF (85). Furthermore, severe systemic hyperinflammation syndromes like HLH can involve the central nervous system (54). The acute necrotizing encephalopathy (ANE), which can be triggered by EBV, may leave survivors with severe neurological sequelae (86).

## 7 Discussion

This review highlights a central clinical dilemma in EBV-associated hepatic failure: the two clinical and pathogenetic patterns — isolated severe EBV hepatitis and EBV-HLH — require fundamentally different treatments; however, these patterns represent ends of a continuous spectrum rather than mutually exclusive categories, and their early clinical presentations often overlap. The key to improving outcomes lies in early differentiation, yet current diagnostic tools have limitations, and the optimal therapeutic approach remains debated.

### 7.1 Pathogenetic concepts and the core clinical challenge

We present the distinction between isolated severe EBV hepatitis and EBV-HLH as a conceptual framework to facilitate clinical understanding, rather than as an established, mutually exclusive classification. The core difference lies in the scope and nature of the immune response, and the two conditions may overlap. In isolated EBV hepatitis, liver injury results from a focused, CD8^+^ T-cell-mediated attack on EBV-infected B cells, with bystander hepatocyte necrosis confined to the liver. In EBV-HLH, by contrast, EBV infection of T or NK cells triggers a systemic hyperinflammatory cascade — a “cytokine storm” involving IFN-γ, TNF-α, IL-6, and IL-18 — with hemophagocytosis and multi-organ involvement. This distinction is not merely academic; it has direct therapeutic implications. Patients with isolated EBV hepatitis generally recover with supportive care, whereas those with EBV-HLH require urgent immunosuppression, and delays in recognition are associated with markedly increased mortality.

However, the clinical reality is more nuanced. Not all patients with EBV-associated hepatic failure fit neatly into one category. Some present with features of both, representing a clinical spectrum rather than discrete entities. The concept of “forme fruste” HLH — where patients meet only part of the HLH-2004 criteria — captures this diagnostic grey zone and underscores the need for a high index of suspicion and a low threshold for initiating immunomodulatory therapy in patients with suggestive clinical features.

### 7.2 Current controversies and differing viewpoints

Several areas of active debate are evident in the literature and clinical practice.

First, the role of antiviral therapy remains controversial. While most evidence supports the view that antiviral agents such as ganciclovir have little benefit in established EBV-HLH — because the liver injury is primarily immune-mediated rather than driven by direct viral cytopathic effects — some experts advocate their use in immunocompromised patients with extremely high viral loads, where reducing the antigenic burden might theoretically attenuate the immune response. However, this approach has not been validated in prospective trials, and a critical principle is that antiviral therapy should not delay the initiation of life-saving immunosuppression.

Second, the diagnostic threshold for HLH is debated. The HLH-2004 criteria were developed primarily for pediatric familial HLH and may not be optimally sensitive for EBV-HLH in adults or immunocompetent patients. Some experts argue that waiting for all five criteria to be met delays treatment and that a threshold of ≥4 criteria — as we propose in this review — may be more appropriate when clinical suspicion is high. Others caution that lowering the threshold risks overtreatment with potentially toxic immunosuppressive agents.

Third, the optimal immunomodulatory regimen is not established. While the HLH-94/2004 protocols (dexamethasone + etoposide) remain the standard of care, there is ongoing debate about whether etoposide should be used upfront or reserved for patients who fail initial dexamethasone monotherapy. Some centers favor a step-up approach starting with corticosteroids alone, while others advocate early etoposide given the high mortality of untreated EBV-HLH. The emergence of targeted biologics — particularly emapalumab, an anti-IFN-γ monoclonal antibody — has further complicated this landscape. While promising in refractory cases, its high cost and limited availability, as well as the lack of head-to-head trials comparing it with etoposide-based regimens, mean that its place in the therapeutic algorithm remains to be defined.

Fourth, the role of blood purification as an adjunctive or salvage therapy is increasingly discussed but remains unproven. While case reports and small series have shown that therapeutic plasma exchange and hemoperfusion can reduce circulating cytokines and, in some cases, viral load, there are no controlled studies demonstrating improved outcomes. The potential benefits must be weighed against the risks of procedural complications and the possibility that removing cytokines might impair the host’s ability to control EBV infection.

### 7.3 Current knowledge gaps and future directions

Several important knowledge gaps limit our ability to manage EBV-associated hepatic failure optimally. Biomarkers are urgently needed to distinguish aggressive immune-mediated hepatitis from early HLH and to guide personalized therapy. Current diagnostic criteria for HLH rely on clinical and laboratory features such as fever, cytopenias, hyperferritinemia, and hemophagocytosis, but these can overlap with severe hepatitis, leading to diagnostic delays (54). Advanced imaging modalities like fluorine-18 fluorodeoxyglucose positron emission tomography/computed tomography (^18^F-FDG PET/CT) show promise, as certain metabolic parameters (e.g., SUVmax of lesions or lymph nodes) can help differentiate malignancy-associated HLH from non-malignancy-associated forms and correlate with inflammatory cytokines such as IFN-γ and ferritin (87, 88). Furthermore, integrating biomarkers such as EBV DNA load into prognostic models may improve predictive performance for outcomes, as seen in other EBV-associated malignancies, suggesting a similar approach could be valuable in hepatic failure (89). The multi-omic signatures, as seen in pediatric ALF investigations revealing a common monocyte-driven inflammatory transcriptomic profile irrespective of the detected virus (AAV2, adenovirus, or herpesviruses), point toward discovering novel, mechanism-based biomarkers (90). Consistent with the narrative scope of this review, no formal meta-analysis or quantitative evidence grading (for example, GRADE) was performed; the evidence strength for key therapeutic and diagnostic points is instead summarized descriptively in **Table 4**.

Clinical trials evaluating the efficacy and timing of novel immunotherapies — such as JAK inhibitors and targeted cytokine blockers — in EBV-associated hyperinflammation syndromes are urgently warranted. The current treatment for HLH, including protocols with etoposide and dexamethasone, is not always effective and carries substantial toxicity (54). The efficacy of immunotherapy in EBV-associated cancers, such as pulmonary lymphoepithelioma-like carcinoma, highlights the potential of immune modulation (89). In the transplant setting, CMVIg prophylaxis has been associated with a significantly lower incidence of EBV infection, a finding attributed not to direct cross-viral neutralization but to its multi-faceted immunomodulatory effects that broadly enhance antiviral immunity, suggesting that similar targeted immunotherapies could play a role in managing post-transplant EBV reactivation and PTLD (82). In ostensibly immunocompetent individuals, primary immunodeficiencies should be investigated, as their genetic and immunological determinants could reveal new therapeutic targets (12).

A higher prevalence of subclinical CMV and EBV DNAemia in patients with ACLF compared to those with stable CLD suggests that these viruses may act as precipitants by exacerbating liver inflammation (5). Investigating the genetic underpinnings — such as polymorphisms in immune response genes (e.g., those affecting monocyte/macrophage activation) that drive fulminant hepatitis, as suggested by transcriptomic studies — could lead to the development of risk stratification tools (5, 90).

The development of a practical, simplified scoring system incorporating readily available parameters — such as ferritin, platelet count, and EBV DNA load — could improve early diagnosis in non-specialist settings. The expanding arsenal of targeted immunotherapies — including JAK inhibitors, IL-1 and IL-6 blockers, and checkpoint inhibitors — offers new possibilities for modulating the inflammatory response in EBV-HLH, although their use in this context remains experimental. Finally, the integration of multidisciplinary collaboration between hepatologists, infectious disease specialists, hematologists, and intensivists is essential, as optimal management of these complex patients requires expertise across multiple domains.

From a global health perspective, the management of EBV-associated hepatic failure is highly resource-intensive and unevenly available worldwide. LT, HSCT, and biological agents such as emapalumab remain concentrated in high-income, specialized centers, while low- and middle-income countries — where EBV infection is ubiquitous and often acquired earlier in life — frequently lack access to quantitative EBV DNA testing, ferritin and sCD25 assays, timely transplantation, and targeted immunotherapies (3, 79). Etoposide and dexamethasone are comparatively inexpensive and widely available, which partly explains their continued central role despite toxicity. The financial burden of novel biologics is a further barrier, and cost-effectiveness data specific to EBV-HLH are absent. Addressing these disparities requires strengthening laboratory capacity, developing affordable biomarkers, and prioritizing the validation of low-cost therapeutic strategies that can be delivered in resource-limited settings.

### 7.4 Concluding remarks

EBV-associated hepatic failure spans two overlapping pathogenetic patterns with divergent treatments, best regarded as a conceptual framework. Early recognition of HLH is life-saving, yet diagnosis remains challenging due to overlapping features and the limitations of current criteria. This review provides a practical clinical framework for differentiating the two entities, highlights controversies and knowledge gaps, and outlines priorities for future research. Multicenter prospective studies are needed to standardize diagnostic algorithms, validate prognostic biomarkers, and establish evidence-based treatment protocols. Ultimately, a personalized approach based on the underlying pathogenetic mechanism — whether immune-mediated liver-confined hepatitis or systemic hyperinflammation — offers the best hope for improving outcomes in this rare but devastating complication of EBV infection.
